# The Intersectionality Between Bi and Multiracial College Students’ Self-identification and Their Behaviors—A Pilot Study

**DOI:** 10.1007/s40615-025-02291-2

**Published:** 2025-02-05

**Authors:** Robert E. Braun, Jade Morant, Margaret Boatright

**Affiliations:** 1 Department of Health and Sport Sciences, Otterbein University, 1 S. Grove St., Westerville, OH 43081 USA; 2 Department of Educational Psychology, Northern Arizona University, Flagstaff, AZ USA

**Keywords:** Bi/Multiracial college students, Health disparities, Racial identification, Health-related behaviors

## Abstract

Due to limited information and published research, health disparities among bi and multiracial (B/MR) groups are not as understood as other racial groups. Without this knowledge and ability to allocate resources as needed, this is another racial group that could suffer from poorer health outcomes. As a result, participants (*n* = 15) were placed in focus groups or individual interviews with ten qualitative questions. Each participant then completed an anonymous quantitative survey assessing their health-related behaviors. Quantitative results included 40% (*n* = 6) of participants who tried cigarettes, 53% (*n* = 8) who tried electronic vapor products, and only 20% (*n* = 3) of participants who got the recommended hours of sleep nightly. Qualitative results include themes of situational identity, White assimilation, and pressure to explain their identity. Many participants dealt with the insensitivity that one side of their family exhibited towards the other side of their identity through inappropriate jokes and comments. Lastly, there were expectations from both family and friends to act a certain way. Researchers identified three major categories that the participant’s influences fell into. Genetics, Culture/Heritage, and the Environment are the aforementioned categories that can work together or stand alone to influence behaviors that can ultimately affect health outcomes. While these results are based on a small sample size (*n* = 15) of undergraduate B/MR students, it does suggest that researchers should complete a more extensive survey on this racial group to verify these findings.

## Introduction

The Center for Disease Control’s National Center for Health Statistics (NCHS) collects, assesses, and publishes data along with the resulting health outcomes for numerous racial groups, including but not limited to Asian Americans, Black Americans, Caucasians, and Hispanic/Latino Americans (see NCHS → Topics → Life Stages and Populations → Race/Ethnicity) [1]. While this is an excellent resource, one racial group is noticeably absent from this list—multi- or biracial individuals. This is especially troublesome since this population will double in size by 2050 [2]. The implications for this racial group doubling in size while not understanding their health needs are quite significant. Without this knowledge and ability to allocate resources as needed, this racial group could suffer poorer health outcomes.

The relationship between race/ethnicity and health outcomes is a topic of ongoing and pertinent discussion within the public health field. Numerous researchers have evaluated health behaviors and links between health outcomes/status and racial identification. The Kaiser Family Foundation (KFF) clearly illustrated that health disparities exist and impact specific communities. For example, Black Americans and Hispanic Americans have higher mortality rates, and are less likely to have access and coverage and receive quality health care [3]. For example, although the Black-White cancer disparity has decreased, Black Americans share a disproportionate burden in deaths for both males and females [4]. According to the CDC, African Americans are not the racial group most likely to use tobacco [5]. However, this group disproportionately suffers more from chronic health outcomes related to this issue, such as heart disease, cancer, and respiratory issues. Alcohol use is another behavior that many individuals perform, regardless of their racial and ethnic groups. According to the NSDUH (National Survey on Drug Use and Health), lifetime alcohol use among all racial groups in 2018 was 83.7% of AI/AN, 70.6% of Asians, 80.2% of Blacks, 78.3% of NHOPI, 91.6% of two or more races, and 92.0% of Whites [6]. However, African Americans are more likely than most groups to experience poorer health outcomes concerning alcohol consumption.

### Background

It is important to note how the history of racism in the USA has affected racial identification, especially for those mixed with Black and White races. Before 1950, the way a bi or multiracial (B/MR) individual identified was not their choice; it was an external decision. The “one-drop” rule, which indicated that a person with any amount of Black blood must identify as Black, was used to reinforce social hierarchy and class systems [7]. However, this rule was so deeply ingrained into the Black community that even when people of mixed race were given the option to identify as B/MR many did not.

It has only been 53 years since the landmark Supreme Court decision of Loving vs. Virginia, which declared interracial marriages as being constitutional in the USA [8]. It was difficult for the USA to acknowledge interracial marriages, let alone their byproducts, being Biracial/Multiracial children. The Multiracial Movement of the 1990s called for a fundamental change to how the federal statistical system classified people by race, calling for the option to choose more than one race in the national census [9]. Due to pressure from the movement, Census 2000 and subsequent federal statistical documents allow for individuals to identify with as many of the listed racial categories as they wish. Because of the knowledge gained from the now available census data, we are able to monitor and predict the rapid growth of this racial group. Kaiser Family Foundation (KFF) acknowledges that the population of those who self-identify as B/MR will double in size by 2050 [3].

Additionally, data collected by national surveys did not account for multiracial individuals when these surveys first started gathering information. This means that those who were self-identifying as biracial or multiracial had to choose an identity for categorization that did not match their intrinsic beliefs. The Behavioral Risk Factor Surveillance System (BRFSS) began in 1984 and added an “other” category in 1985. Only in the 2001 survey did individuals have the choice to choose “one or more of the following…” [10]. Its counterpart, the Youth Risk Behavioral Surveillance System (YRBSS), started in 1991 with an “other” category but only started with their 1999 survey allowing individuals to “select one or more responses” for their race question [11]. Based on these surveys, it seems B/MR individuals, specifically adolescents, were the “forgotten” population. Though there have been efforts to correct this, this population often continues to receive little acknowledgement as its own subset of the B/MR population.

### Existing Literature Regarding Identity Development

The identity of multiracial individuals is fluid and affected by internal and external factors. For college students, identity development continues through both public and private spaces. Public spaces include residence halls, student organizations, classrooms, and social events. Private spaces include journaling, academic projects, or conversations with trusted others [12]. Social constructs affecting identity and identity development include gender, social class, family, community, peers, age, spirituality, social awareness, cultural awareness, and geographical region [13]. A quote from Grillo states, “When an adolescent begins to question who they are, this process often begins with who they are in comparison to other people” [7p431].

Although ethnic identity is a cultural component and not a biological one, like racial identity, this sets an important framework for B/MR individuals who blend both biological and cultural components in order to discover their sense of self. Jean Phinney, a psychologist who has a great deal of work focusing on social psychology and ethnic groups and ethnic identity development, as well as identity and developmental psychology, has developed a 3-stage model that is commonly accepted for ethnic identity development [14]. Phinney defines ethnic identity in three stages: “(a) commitment and attachment—the extent of an individual’s sense of belonging to his or her group; (b) exploration—engaging in activities that increase knowledge and experiences of one’s ethnicity; (c) achieved ethnic identity—having a clear sense of group membership and what one’s ethnicity means to the individual [14p3, [15].” For individuals who identify as B/MR, ethnic identity development is further explained by Poston’s Biracial Identity Development Model, which includes a progression through the following stages: (a) Personal Identity; (b) Choice of Group Categorization; (c) Enmeshment and Denial; (d) Appreciation of Multiple Identity and Exploration of Heritages; (e) Integration and Valuing of Multicultural Identity [16]. Furthermore, multiracial individuals tend to feel between two (or more) identities instead of feeling accepted in all parts of their identity. This can lead to feelings of not belonging, creating stress that can affect one’s health. These feelings of being stuck between two identities tie into both the marginal man theory and double consciousness as coined by WEB Du Bois [7, 17].

There are a variety of societal issues that are unique to this population, especially on college campuses. An article written by a multiracial woman named Allison King highlights her experiences in college dealing with identity and the experiences of other biracial or multiracial college students she included in her study [12]. Her study’s results included major themes around issues with a sense of belonging, having the inability to exist and figure out their identity as they grew. Feelings of alienation or loneliness, trying to balance identifying with both or all parts of their identity, peer pressure to choose one social group over another or being forced into one group based on appearance, discomfort in their identity, or feeling invisible were also themes present in her research.

The B/MR community is also forced to face the unique issue of monoracism, which is a variant form of racism that prioritizes privileging monoracial identities. This concept contributes to the erasure of multiracial people’s experiences. On an institutional level, monoracism has shaped preferences towards the conceptualization of race to be viewed in mutually exclusive terms and categories [18]. Monoracism presents its effects in many forms, including monoracial student recruitment and retention centers on college campuses, electronic medical databases and record systems that are programmed with the inability to handle more than one race selection at a time, multiracial people needing to defend their self-identifications, healthcare professionals making inappropriate assumptions or comments based on physical appearance, or TSA workers flagging parents of multiracial children for potential trafficking because their children may not look like them, or even simply not having an opportunity to choose more than one race when filling out documentation [18].

Another set of issues revolves around lacking the cultural tools to be accepted into a certain group; tools including language, experiences, or cultural knowledge [12]. The participants also experienced social exclusion from peers from one or another racial group that the student identifies with. In addition, multiracial students struggle from a lack of individual or organizational representation on their campuses. These students lack safe spaces on their campuses where they are not forced to conform to just one part of their identity. Recommendations for improving acceptance in the college setting for multicultural students from King include having open conversations about multicultural identity, creating organizations for multicultural students, and better representation of multiracial students on campus (including faculty and staff) [12].

Previous studies implicate further health research on the B/MR population. Structural and conceptual frameworks suggest various factors that affect one’s identity and, as a result, their behaviors and health outcomes. Both race and socioeconomic class jointly contribute to health risks. For B/MR individuals, stress has been identified as a critical mediator in their health outcomes as it is related to their identification. This is especially true when one’s identity is misaligned with the outside perception of their identity, which is a common issue within this population [7]. Often, when one’s self-identification does not match their physical appearance, it can lead to feelings of disconnectedness and lower levels of psychological well-being [7]. Furthermore, researchers analyzing data from the Adverse Childhood Experience Questionnaire discovered multiracial individuals having higher reported rates of anxiety than their monoracial peers [19].

For multiracial individuals, imposter syndrome can affect identity. Jennifer Cheang, a Chinese and Puerto Rican author, states, “I’ve always been considered “half” or “watered down” versions of my Chinese and Puerto Rican identities [20].” This sense of not belonging makes one feel like an imposter in their own identity. Issues she notes are faced in the multiracial community include colorism, exclusion, isolation, lack of representation, privilege, and finding healing [20]. Though many health findings indicate that biracial individuals experienced poor mental and physical health outcomes. Other findings indicate that B/MR individuals who have a positive relationship with their identity are linked to good psychological health including higher self-esteem, increased efficacy, and decreased stereotype vulnerability [13].

Researchers analyzed data about young adult college students collected by the Healthy Minds Study in one study. Their findings indicate that multiracial college students, compared to their monoracial peers, experienced higher rates of mental and behavioral health issues, including depression, anxiety, languishing, perceived need for help, loneliness, and drug use (*p* < 0.001). This group also presented an increased likelihood of having psychiatric disorders as compared to their monoracial peers. Psychiatric disorders, including depressive disorders, bipolar disorders, trauma (stressors) disorders, neurodevelopment disorders, eating disorders, and personality disorders, were all more prevalent within the multiracial student sample than for their monoracial peers (*p* < 0.029 or less). Findings within this study presented modestly greater odds of all mental and behavioral health outcomes to occur for multiracial individuals [21]. This adds significant information to the conversation surrounding B/MR college students and health behaviors, as these issues are significantly impacted by environment, identity, and societal acceptance. As we learn more about this racial group, we hope to uncover the intersectionality of self-identification and health behaviors within this population.

In a study comparing the health risk status of mixed-race adolescents between the 7th and 12th grades with their single-race counterparts, mixed-race individuals were found to have a higher prevalence in areas such as poorer general health, waking up tired, skin problems, headaches, aches and pains, sleep problems, depression, regular smoking, regular drinking, and being regularly drunk [22]. Additionally, mixed-race adolescents were also within the boundary values for the non-risk individual and family attributes of the single-race groups that constitute their identities, such as student GPA, family structure, and family education [22]. Meaning, these student scores were found to lie between the norms of their family’s monoracial identification. Furthermore, this corresponds with research indicating that for studied health-related behaviors (smoking, sexual health, and alcohol use), B/MR individuals fall between the norms of their monoracial peers, when performing these behaviors [23].

For multiracial adolescents, situational identities are often formed where the individual will shift between categorizations, using whichever serves them the best at that time. This is an example of code-switching. The American Psychological Association defines code-switching as “the practice typical of individuals proficient in two or more registers [24].” With the fluidity of multiracial identity, we find that B/MR individuals may choose to self-identify differently in different settings or times in their lives [18]. Though establishing situational identity can be helpful in the short term, studies have shown that higher self-esteem and higher socioeconomic status are associated with lower rates of moving between racial categories [13].

### Research Question

We have established notable health and social implications for the B/MR community influenced by their race. Social implications include feelings of not belonging, stress, alienation, loneliness, invisibility, disconnectedness, imposter syndrome, and lowered psychosocial well-being. Health risks such as poorer general health, waking up tired, skin problems, headaches, aches and pains, sleep problems, depression, regular smoking, regular drinking, and being regularly drunk have been studied in Udry’s research [22]. Conversely, there is research to support positive relationships between multiracial heritage and health when there is a positive relationship with one’s identity. This is supported by Renn, who linked a positive relationship with multiracial identity to higher self-esteem, increased efficacy, and decreased stereotype vulnerability [13]. The lack of mass empirical research on self-identified multiracial individuals, specifically multiracial college students, has led a research group at a small Midwest liberal arts college in Ohio to explore this population. B/MR individuals are an increasing demographic, which calls for increased literature and research that can be used to help identify the needs of this population, especially within the public health space. In the following study, researchers seek to explore the attitudes, beliefs, and factors that affect their health. We aim to answer the question, “What is the intersectionality between undergraduate college students who self-identify as Bi or Multiracial (B/MR) and their behaviors?”.

## Methods

### Participants

Fifteen undergraduate students participated in this study. Thirteen identified as female, one as male, and one as “other.” Racially, ten self-identified as multiracial while three identified as “other,” one identified as Black/African American, and one chose three options. Six identified as seniors, four identified as freshmen, and three identified as sophomores. Regarding their parent’s racial identity, two-thirds of their mothers are White/Caucasian, while one-third of their fathers identify as Black/African American.

### Procedure

The study took place at a small Midwest liberal arts college in Ohio. The researchers obtained a list of self-identifying B/MR students from the university’s institutional advancement (*n* = 124). Afterward, all students included on the list were emailed to encourage registration for the study. Study participants were recruited via email and through signs posted around this university’s campus. As students registered, the research assistants attempted to place them in a focus group based on availability. To further encourage participation, the target population was offered a $20 gift card to the Meijer grocery store if they became involved in the study.

Due to scheduling conflicts, the principle investigator and three research assistants ran four focus groups and three individual interviews independent of the focus groups. Each focus group included 2–5 students structured with ten questions asked in each session by the principle investigator. The principle investigator conducted all sessions, which were also recorded for clarity using OtterAI [25]. The research assistants took notes for thematic responses throughout the interview and focus group sessions. After participation in the focus groups, each participant was asked and completed an anonymous survey with 48 questions to assess their health-related behaviors. This was done in order to gain more intimate and sensitive data about each participant’s behavior.

### Instrument

While 10 questions were asked during the focus groups/interview sessions, 6 of those questions were assessed in this paper (see Table 1). A total of 48 questions were included in the survey, of these 38 questions came from the YRBSS (Youth Risk Behavioral Surveillance System). Lastly, four questions from the CDC Health-Related Quality of Life (HRQOL) assessment were included [26]. Overall, questions included in the survey covered behaviors such as smoking/vaping, sexual, alcohol use, nutritional habits, sleep behaviors, and demographics. Most questions were nominal or ordinal, closed-ended, and “forced” the participant to select an answer.

**Table 1:** Focus Group/Interview Questions used in this analysis

**Question 1**: Self-identification (who you are vs. who people perceive you to be). Have you had any issues in the past with self-identification and please describe
**Question 2**: Are you asked to choose one race to identify with? Why do you think that happens?
**Question 3**: How would you describe your environment growing up? Was it more diverse or monoracial? What opportunities did you have, etc. Did you feel that you were a minority within your own community? Why or why not?
**Question 4**: How healthy are you if you are comparing yourself to your peers? Has your race affected your health or health outcomes in any way? If you feel you are unhealthy what has caused you to feel this way? Do you feel you’re more risky with your behaviors than your friends that you hang out with?
**Question 5**: Do you feel you should act in a certain way because you are B/MR. Do you feel peer pressure and if so where do you think the source of that pressure is coming from?
**Question 6**: Do you think your appearance/racial features affect your day-to-day life/well-being?

### Data Analysis

For the quantitative data, the information was coded, cleaned, and inputted into SPSS (Statistics for the Social Sciences), version 26 [27]. Descriptive analysis was completed to assess means, percentages, and counts for the 15 surveys that were completed.

For the qualitative data, OtterAI software was used to download and convert the content of the interviews and focus groups to Microsoft Word [25]. The findings were then reviewed by researchers and cleaned up to ensure the typed version matched the audio recordings. Thematic analysis was conducted among the research team and recurring themes were pulled from the interview and focus group results. This process was completed by three members of the research team individually and themes were discussed in a group setting to identify the most significant recurring discussion points.

## Results

### Quantitative Analysis

#### Overview

For the quantitative survey, participants answered questions about their health behaviors. Health behaviors analyzed include alcohol consumption, sexual health, healthy days, sleep, vaping, smoking cigarettes, fruit and vegetable consumption, and whether or not they have seen a dentist in the past 12 months. Table 2 shows many of the behaviors assessed in this survey by frequency and percentage. Other behaviors, such as alcohol, sexual health, and healthy days, are summarized in the following sections.

**Table 2:** Selected Behaviors among B/MR Participants (*n* = 15)

Behavior	n	%
Sleep 8 + hours/night	3	20
Cigarette Smoking (ever)	6	40
has smoked in the last 30 days	2	13.3
Vaping (ever)	8	53.3
has vaped in the last 30 days	3	20
Saw a dentist during the past 12 months	10	66.7
Eating green salads at least twice/day	3	20
Eating fruits at least once/day	7	46.7

#### Alcohol Consumption

Overall, 15 participants answered questions related to alcohol consumption. They were first asked about their age when they first consumed alcohol (other than a few sips), and 60% of the participants (*n* = 9) were above the age of 17, 26.7% (*n* = 4) were 15 to 16 years old, and others answered from 11 to 14 years old. The participants were then asked how many days they had at least one drink of alcohol during the past 30 days. The highest reported answer was 1 to 2 days (53.3%; *n* = 8), while the next highest reported response was 10 to 19 days (20%; *n* = 3). Lastly, participants were asked if they binge drank in the last 30 days. Most reported that they did not (53.3%; *n* = 8), while three reported binge drinking one day (20%; *n* = 3). One respondent binge drank from 10 to 19 days in the past 30 days.

#### Sexual Health Information

Participants answered a series of questions about their sexual health behaviors. Overall, 66% (*n* = 10) reported having sexual intercourse. Twenty percent of participants were between twelve and 16 years old, 13.3% were between seventeen and eighteen, and 33.3% were over eighteen. Twenty percent of the remaining participants drank alcohol or used drugs before their most recent sexual intercourse. Sixty-six point seven percent of participants who reported having sexual intercourse used a condom their last time. Only 40% of these participants have ever tested for HIV. However, 60% were tested for other sexually transmitted diseases other than HIV.

#### Healthy Days Information

Participants were asked about their healthy days over a 30-day period using the Health-Related Quality of Life (HRQOL) questionnaire [26]. The first question assessed the general health of the participants, with the options being “excellent,” “very good,” “good,” “fair,” or “poor.” Of all participants (*n* = 15), 6.7% answered “excellent,” 13.3% answered “very good,” 46.7% answered “good,” 33.3% answered “fair,” and there were no responses for “poor.” The second question assessed physical health, including physical illness and injury, and how many days during the last 30 days the participants had poor physical health. The mean answer (*m* = 5.2 days) indicates that the participants had poor physical health on average, just under 1 week for the past month. The third question states, “Now thinking about your mental health, which includes stress, depression, and problems with emotions, for how many days during the past 30 days was your mental health not good?” The mean answer (*m* = 15.7 days) indicates the participants’ suffered from poor mental health over 2 weeks for the past month. The final question about healthy days states, “During the past 30 days, approximately about how many days did poor physical or mental health keep you from doing your usual activities such as self-care, work, or recreation?” The mean answer (*m* = 8.1 days) indicates that the participants were unable to perform their normal functions for just over a week within the past month due to poor health.

### Qualitative Analysis

#### Question 1

Themes identified in the responses include ideas of struggling to fit in or find a community, finding pride in both parts of their identity, and lack of inclusion in an array of categories, including a lack of racial representation on questionnaires/surveys, and lack of having B/MR options to choose from on federal or legislative documents. Bi and multiracial students report having experienced struggles to fit in or find a community of other individuals with similar experiences. Identifying as multiple racial identities, as opposed to one, means there is a broader scope of experiences that the individual may identify with, and there is also an experience of being bi or multiracial that only other members of this community can identify with. Subjects also reported feelings of pride in both or all parts of their identity. This comes with advocating for both or all aspects of their identity and often acting as a mediator between the opposing parts of their identity. The participants also found themselves lacking inclusion (racially) in several spaces, including on questionnaires or surveys, and a lack of federal or legislative consideration. Examples of this include the lack of an option to choose multiple races on demographic questions or being forced to identify as “other” if they do not want to choose just one part of their identity.

#### Direct Quotes

- …it’s like part of you wants to say mixed and then describe why but they don’t give you that option
- As you get older it’s easier to blend. But then there’s that whole like thing of fusion food and like fusing your two cultures is blasphemous because it’s not the original pure form of it. And so, when there’s all that rhetoric about fusion cultures not being it not being right not being good enough. Then you feel like you’re not right not pure not good enough because you’re the same.
- I guess like sometimes, they ask you, like how you identify, I’ve had times where always frustrates me because I don’t want to pick between the two because I’m both I’m not just one” they don’t give you an option like for multiracial or anything it’s just black, white or Hispanic or Asian, when there’s not like another box or you can’t pick more than one and that

#### Question 2

Within the responses, there were three themes identified throughout the focus groups. The first theme identified was participants’ difficulty embracing one part of their racial identity. This was both an internal and external struggle for participants. Internal struggles of embracing identity include not feeling part of the identity based on values, culture, language, etc., whereas some may encounter physical struggles, including a lack of features associated with a particular part of their identity. External struggles included their family and friends’ expectations for them to act a certain way based on their preconceived beliefs about their racial identity. The second theme participants shared was being forced to check one box regarding racial identity on applications or documents. For example, one participant talks about the frustration of not having the opportunity to identify as multiracial, forcing them to side with only one part of their identity. This is important for identity development and recognition, because, in these scenarios, they are only allowed to fully embrace one part of their racial identity, forcing them to either consciously or subconsciously prioritize one side over the other. The last theme highlighted from this section is the highlight of and exposure to only one side of the participant’s race within their home environment. This is especially true for the minority part of their identity, as many participants come from monoracial communities. These participants primarily grew up in White neighborhoods and were not exposed to the other dynamic of their racial identity outside of their homes.

#### Direct Quotes

- …a lot of my friends, like my really close friends who are also multiracial… that I have more in common with people, even if they’re a completely different mix than I am, who are mixed than I do with either my like identities…
- …sometimes, they asked you, like how you identify, I’ve had times where they don’t give you an option like for multiracial or anything it’s just black, white or Hispanic or Asian, when there’s not like another box or you can’t pick more than one and that always frustrates me because I don’t want to pick between the two because I’m both I’m not just one

#### Question 3

Themes from the respondents include participants in this study coming from mainly monoracial communities. The most common experience was attending a monoracial (majority White) school and the community environment where they grew up. However, many were exposed to a diverse environment at some point in their lives. Still, most participants identified themselves as a minority in their community. On the other hand, these participants also reported a positive relationship with access to health experiences such as community centers and opportunities for physical fitness, healthy eating, and health insurance.

#### Direct Quotes

- “Yes, that’s a very easy answer. I have always felt like a minority in both groups. I don’t ever feel completely white and I don’t ever feel completely brown. And I always feel just a little bit off hanging out with like, white people or brown people. It definitely helps that a lot of my brown friends are born and raised here. So they have been like, pretty whitewashed anyways. But if I’m in a if like I ever visit Pakistan, I feel very different.”

#### Question 4

Participants felt that they had no significant differences in health compared to their peers and, for the most part, felt healthy. Those who performed fewer risk-taking behaviors than their peers were directly linked to influences from their home life. Some were afraid to participate in riskier behaviors due to a need to hold a specific image because of their race/ethnicity or culture. However, many participants were exposed to chronic, hereditary health conditions or diseases based on their family history. From their viewpoint, it seems their health was more affected by their family’s history (genetics) than by culture or heritage, while acknowledging their behaviors (for some) are influenced to an extent by their cultural upbringing. Ultimately, through this discussion, being who they are (i.e., the B/MR social classification) did not affect their health or health outcomes. Rather, their cultural influences and the genetics inherited from their parents played a more decisive role in their health.

#### Direct Quotes

- I would not say I’m healthier, like, as my peers like we’re probably about the same. And yeah like both my parents have major health issues, on both sides… so like, either way I’m predisposed to a lot of things, and I’ve just kind of accepted that.
- I would say I would do just as much as my, my peers do I don’t do any more, any less but I do try to watch on my dad’s side of him and his brother pretty much all his cousins, all the males on my dad’s side are big, big potheads. So I try to like stay away from that one but I think I do just as I would say just as not as risky as my friends will be. I don’t want to be overly risky
- …checking white on the little box saying that I was a white person gave me a lot of privilege in a doctor’s office.

#### Question 5

Participants responded that they maintained situational identities triggered by different groups of people. They also felt the need to code-switch within their career due to pressures to act “more White” or assimilate towards “Whiteness” to make friends or build professional relationships. Furthermore, B/MR students perceive societal norms as leading to shared stereotypes from family, friends, and the community. These social structures also pressured these participants not to portray the stereotypes of their minority cultures. However, there is a certain level of expectation to maintain the minority culture and play into some stereotypes within certain familial and social groups. These participants had to walk a tightrope between their identities, having nowhere (i.e., a safe space) to be their true selves.

#### Direct Quotes

- I’m not trying to be somebody who I’m not, you’re just trying to not let me be somebody who I already am.
- I’m kind of like a foggy mirror. I reflect whatever people I am around, but I always have to keep in mind that there are other things behind me, and I’m a little bit different.

#### Question 6

Participants noted their worries about the perception of others because of their race. The racial feature most frequently mentioned by participants was hair. Besides their hair, their lips, eyes, and nose were mentioned as frequently identified racial features that were brought up in their daily life. Many participants drew upon early recollections of comparing hairstyles and textures between themselves and their family, friends, and peers. One student noted differences between her and her mom’s hair texture and how that affected her outlook on her hair. It took acceptance of her own hair to come to terms with that part of her identity. The participants also noted the effects of racism in professional settings, the workplace, and interviews. An example of this would be hair in the workplace and the expectation for it to be straightened or pulled back if they have a naturally curly or kinky hair texture. Participants report on the duality of their feelings towards the conversation around their hair. On one side of the coin, a particular participant reported annoyance related to individuals commenting on her Black features. In contrast, her Black features made her feel more connected to the Black community. The duality of frustration versus validation was a common theme among participants.

#### Direct Quotes

- My hair was always like my big thing like it took me probably until end of high school to like come to the fact of like well, I have curly hair, and you’re just gonna have to deal with it… it takes me out to put all this product in my hair, I have to do this and I have to do that like a good 30 min my morning might just be scheduled to my hair, and all of my white friends are all like, oh I just like wake up and like put a pin in it and then go teach, and I’m like, wait, I can’t, I wish!
- …people have also commented on my lips because my lips are bigger than my mom’s and like they’re more like black features I look just like my dad because my lips and my nose and my eyes, so people comment on them like, I wish I had big lips like yours I’m like… but some black people have been able to tell I blacked by those features and that makes me feel seen in a lot of ways, but I’m also like, are we also stereotyping ourselves in doing that.
- I don’t think it’s like outright external that’s coming from the community as much it’s just internal internally. Having that stress and everything of what you’re thinking other people think due to the perceptions of like the media and stuff that we grew up with.

## Discussion/Conclusions

**Fig. 1:**
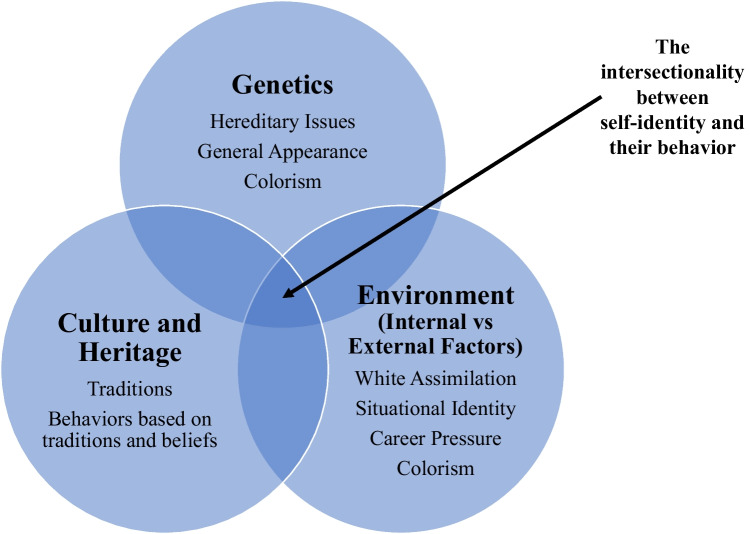
B/MR Conceptual framework (three major themes in intersectionality between identity and behavior)

Throughout the analysis of these results, researchers discovered three major themes that have the capacity to stand alone or work together to help explain the behaviors of members within the B/MR community. The influences of genetics, culture/heritage, and the environment are all contributing factors in determining the behaviors which can affect health outcomes. Figure 1 demonstrates this conceptual framework as a Venn diagram with three overlapping circles each containing distinct constructs that can be used to predict the influences on behaviors of an individual, especially as it relates to health within this population. Furthermore, these three concepts fit nicely within Bronfenbrenner’s Ecological Systems Theory [28]. To further explain, the genetics, culture/heritage, and environment constructs revealed in this research fit within Bronfenbrenner’s microsystem level. In contrast, the interaction among these three constructs, which can lead to a particular behavior, falls within the mesosystem.

As indicated by Renn and Smith, “Bronfenbrenner’s (1977, 1995) bioecological systems model is one of the most widely used in studies of human development” (p. 13) [29]. The model proposes four features of an ecological system that characterize the setting in which human development occurs. These features are as follows: Person, Process, Context, and Time (PPCT). Person refers to the developing individual and their unique characteristics. Context includes four nested ecological systems (the microsystem, mesosystem, exosystem, and macrosystem). Process involves interactions between the person and their ecological systems. Time represents lived experiences, historical events, and societal changes that span a person’s lifetime.

Findings from this study indicate that B/MR college students’ genetics and culture/heritage and environment have significant influence in determining health-related behaviors and outcomes. The current findings can be interpreted through the lens of Brofenbrenner’s bioecological systems model where genetics is recognized as a characteristic of the person while cultural belief systems and heritage are components of the macrosystem.

Additional findings from this study reveal the impact of environment on college students’ health-related behaviors and outcomes. Renn’s model of Biracial and Multicultural identity expands Bronfenbrenner’s ecological theory and identifies five patterns of identity among multicultural college students, several of which are represented in the current study: (1) Student holds a monoracial identity; (2) Student holds multiple monoracial identities, shifting according to the situation; (3) Student holds a Multiracial identity; (4) Student holds an extraracial identity by deconstructing race or opting out of identification with US racial categories; and (5) Student holds a situational identity, identifying differently in different contexts [30, 31]. Evidence of these patterns of identity has been noted through the current analysis, thus further validating Renn’s findings [30, 31].

### Genetic Influence

There are numerous ways that genetics impacts identity, behavior, and health. Throughout the discussions, the participants mentioned specific genetic issues, such as familial histories of mental health and chronic conditions or illnesses. These were discussions that were not planned for but contributed to a significant theme nonetheless. All of the aforementioned examples did influence the participant’s behavior; however, the one genetic issue and theme that seemed to resonate the most pertained to the way a person looks. A recurring theme within the responses includes the idea of being White passing and the privileges that come with that, due to the fairness of their complexion. White passing is when someone perceives a BIPOC person (Black, Indigenous, and People of Color) as a White person, for whatever reason [32]. The privileges that accompany this trait include safety and opportunity in areas like travel, education, and employment [33]. However, there are also some disadvantages identified by participants that come with White passing. One of which is the idea that lacking features commonly associated with a cultural or racial group leads to imposter syndrome when making cultural choices. This can lead to a B/MR individual feeling the need to explain oneself for choices that they are entitled to make. These appearances lead to feelings of either validation or invalidation, and how the participants feel about themselves racially, or within their skin.

The intersection of genetics for college students who self-identify as B/MR contributes to their behaviors in a variety of ways identified throughout the results. General appearances for B/MR college students lead to behaviors of assimilation to avoid being stereotyped or to fit in as identified by our participants. General appearances also allow for passing privileges which students identify as a resource used to make advances or have benefits that they otherwise would not be afforded. One student gives an example of this where they state, “…checking white on the little box saying that I was a white person gave me a lot of privilege in a doctor’s office.” Another intersection between genetics and behavior for this population is the comparison between the features of themselves vs. family, friends, and peers. In one example, a participant compares her curly hair to her mom’s long pin straight hair and expresses how that was all that she ever wanted as she was growing up and how it has been a continual struggle throughout her life to accept features such as hair type that differed from her mother and peers around her who also had straight hair.

Participants also expressed frustration and or validation based on their general appearance or racial features. This occurs when one’s more ethnic features are continually pointed out like curly/kinky hair. It is frustrating as they may not want people talking about their features or invading their personal boundaries by doing things such as touching their hair. However, these same features allow them to feel closer to other parts of their identity through their shared feature or features. Lastly, general appearance can create pressure to explain identity to others if their looks do not fall within the stereotypical features of their racial demographic. This has caused some participants to make decisions that they otherwise would not have made if their outer appearance matched their self-identity.

### Cultural and Heritage Influence

Culture and heritage also demonstrated a prominent influence on identity and behavior, since many participants mentioned that the culture and heritage they were aware of and grew up knowing played a role in their behavior. Frequently, their behavior was dictated by what they knew about one side of their racial makeup. For example, nutrition (what they eat) was based for some on their cultural upbringing, extending into their college years. The same went for alcohol and tobacco use.

This is important because B/MR children often have different cultural experiences than their family members. Oftentimes, members of this community are celebrated for muted culture on one side of their family and ridiculed for it on the other. Whether it is their lack of knowledge about the history, language, heritage, and culture on either side of their racial identity, or attempting to assimilate towards one side or the other, participants recall frequent ridicule and labels of being “washed out,” “watered down,” or incomplete versions of either side of their identity. Cheang, who is Chinese and Puerto Rican, stated, “I’ve always been considered “half” or “watered down” versions of my Chinese and Puerto Rican identities [20p2].” This sense of not belonging makes one feel like an imposter in their own identity. Her findings found that this led to issues that the B/MR community face, which includes colorism, exclusion/isolation, lack of representation, privilege, and finding healing [20]. Furthermore, the participants did not mention having the opportunity to learn more about the other side of their identity, nor did their families go out of their way to educate them. This leaves these participants feeling less culturally competent or belonging than their monoracial peers from either side of their identity. One participant was quoted stating, “I felt like our home experiences [were] pretty monoracial because the only real culture that’s existing here is brown culture.” Explaining that the White part of their racial identity does not necessarily have a culture nor was that culture discussed at home. Rather, they fed off American culture. Unfortunately, this can and frequently did lead to reported situations of gaslighting and hostile environments from one side of their family as it related to the other side of their racial identity from the participants within the study.

Bi and multiracial individuals, no matter their genetic makeup, identify as having a common culture more similar to one another’s experiences than either/any of their monoracial sides of their identity. This was an overarching theme throughout this study, and one that other studies identified as well [33, 34]. A participant stated, “…a lot of my friends, like my really close friends and I still have and have had for a long time are also multiracial, and that I have more in common with people, even if they’re a completely different mix than I am, who are mixed, than I do with either my like identities…”.

For B/MR individuals, partaking in behaviors that are directly linked to their home life, cultural upbringing, or cultural practices have been identified throughout the research. An example of cultural practices that influence health behaviors is nutrition. With being introduced to one, both, or all parts of their cultural cuisine, there are different nutritional values or elements that are stressed within particular cultures. The blending of cultural foods was also mentioned by one participant who suggests that fusing cultures (like food) results in a loss of authenticity. Another intersection between this population and culture/heritage is knowledge or lack thereof regarding history, language, heritage, and culture which leads to behaviors that may or may not reflect the norms of one racial identity or the other. A lack of education about one’s culture creates a critical information gap. For individuals who have never been educated regarding this matter, they have no way to know the expectations of the identity they are expected to assume. Lastly, for B/MR individuals, there is evidence of an established common culture with other B/MR individuals that is more similar than the experiences of either part of their monoracial identity. The behavior of increased correspondence, friendships, and attraction to others who come from two or more racial identity additionally allows these individuals to learn and share their experiences that are both common and different with one another.

### Environmental Influence (Internal vs. External Forces)

Environmental influences, both internal and external, also represent a major set of themes in this study which can influence identity, behavior, and health. A major environmental influence includes assimilation towards Whiteness while attempting to mask their minority culture. This could occur conscious or subconsciously, and by design, either internally or due to outside factors. This was reported due to career pressure to “act whiter” in order to make friends, build relationships professionally, or climb the corporate ladder. An example often portrayed is through their hair which was a common theme among participants [35]. This issue, unfortunately, is something introduced to children of color far before they enter the workplace. Over a decade of national research indicates that girls of color are most likely to be disciplined in school for the ways they wear their hair, whether that be through hair as self-expression, culturally rooted hairstyles, or hair coverings. The option to choose how to wear their hair is a natural and important part of healthy adolescent development in young girls of color [36].

Though there was pressure towards assimilation, there was also an expectation to act like the stereotypes based on their minority identity. These external pressures cause the participants to struggle internally with not only being their full authentic selves, but even identifying what their most authentic self is. This leads to code switching or situational identities triggered by the societal or specific expectations of others. Basically, the external factor here is the stereotype or expectation of an individual other than one’s self and the internal factor is the participant’s desire to “fit in,” be “liked” or “accepted.” Because of this, one’s desire to fit in, be liked, accepted, or even understood would lead to assimilation towards a more palatable identity. Most participants did discuss how significant a role code switching played in their day-to-day life, and how it helps them cope with and successfully move between friend groups.

Conversely, participants often felt that they had no space to be themselves. One participant uses the analogy that she is like a foggy mirror that reflects whoever she is around. This is to amplify that her own identity is shifted and manipulated by her surroundings, implying that she has no true sense of her own identity as she has always had to reflect what she was around in that moment. The ultimate question is can they ever truly be themselves? Some said they never had that chance, and that it is exhausting always being someone else without ever having the opportunity to explore who they really are foundationally.

Participants also reported feelings of pressure to explain their identity to others, especially when their outer appearance does not match their personal identification. Their looks lead to feelings of validation or invalidation about how they feel racially, and often increased pressure for explanation. For B/MR individuals, stress has been identified as a critical mediator in their health outcomes as it is related to their identification throughout numerous studies [7, 22].

This has been proven to be especially true when one’s self-identity is misaligned with the outside perception of their identity proving problematic as this is a common issue within this population and can lead to feelings of disconnectedness and lower levels of psychological well-being [7]. For multiracial individuals, imposter syndrome can affect identity.

Other prime examples that influence the way that this population perceives their identity are questionnaires and documentation that do not give the option to choose more than one race, discounting their complex identity, which is not able to be categorized into a singular racial identification. When documents ask questions related to demographic information, yet do not have the appropriate options to report all races, it belittles the identity and experiences of those who live outside of the monoracial social classification. This issue was discussed many times throughout the focus group and individual interviews.

Overall, the intersection of the environment for college students who self-identify as B/MR contributes to the behaviors of this population in many ways. For B/MR individuals, being forced to choose a single racial identification in certain environments is an identified behavior that all participants have experienced. Situational identities triggered by different groups of people were another common experience between participants. Societal norms have also been identified as leading to shared stereotypes from family, friends, and community, which contribute to behaviors that can be as simple as choosing whether or not to wear cultural clothing, participating in clubs or organizations that are designed for one part of their identity or the other, how to wear their hair, whether to code-switch, and who they may code-switch around. These examples lead to an internal vs. external struggle in finding one’s identity if an individual sees themselves as one thing and society views them as another. Because of this, students often straddle a line between their own expectations of themselves and those that others place on them. Lastly, behavioral expectations based on stereotypes related to their minority identity are a strong external factor. This is true for whichever racial group is the minority in the environment, even if it is the societally bigger group.

### Interactions Among Genetics, Culture, and Their Environment

As demonstrated in Fig 1, all of these factors play a role together in forming one’s identity and resulting behaviors, particularly as it relates to their B/MR identity. For example, the intersectionality of identity and behavior as it relates to colorism can be found between genetics and their environment. Genetic makeup contributes to how an individual looks externally, and the *external* environment judges and stereotypes individuals based on their looks. Culture and heritage find an intersection with the environment when behaviors that are based on traditions and cultural beliefs are impacted by White assimilation. It can be difficult to maintain cultural behaviors, especially from a minority group, in the midst of attempting assimilation towards Whiteness, in order to have an easier time entering the workforce through internships, and beginning to work in their fields. The intersectionality between genetics with culture and heritage can relate to general appearance and behaviors that have roots in tradition, or if an individual will be accepted into the culture based on their appearance. This can include cultural attire, as well as societal issues such as colorism. Lastly, there are certain expectations of physical appearance within certain racial, cultural, or ethnic groups. These can be harmful to B/MR individuals as their physical appearances may not align with members of either group that contributed to their genetic makeup, making it more difficult to be or feel included in traditions of their culture or heritage. Ultimately, these three concepts can work together or independently of one another in order to influence or affect the behavior of a person who identifies as B/MR.

### Future Considerations

Attempting to address and eliminate these issues is no easy task. Based on this research, the authors have some suggestions to alleviate the pressures that those who self-identified as B/MR face. For one, colleges could be more cognizant of these individuals’ issues, possibly by offering support groups or having a student group specifically for B/MR students. Additionally, offering counseling or having a counselor who resembles them or mirrors their experiences would be helpful. Colleges and universities should be intentional, specific, and mindful of their hiring practices. They should be specific in their advertisements to attract faculty, staff, and administration of color to represent their student population better. University employees should reflect the student body population they serve. College personnel should also seek their opinions to ensure their voice is included and heard regarding issues B/MR college students face. Do not just assume that their voice is heard. Be intentional when collecting data, and make sure they are included in the discussions. Researchers can also focus on the ACHA/NCHA longitudinal study and run analyses over time focusing on B/MR college students to better understand their behaviors. The same analysis can be performed using the YRBSS survey for B/MR youth and high school students. Lastly, future research should include more colleges and universities, such as HBCUs (Historically Black Colleges and Universities) and PWIs (Predominantly White Institutions), which include public and private institutions, when assessing B/MR college students, regardless of methodology.

### Limitations

As positive as the results of this study are, some limitations need to be acknowledged and clarified. The first is within this study, researchers conducted four focus groups and three individual interviews. Acknowledging that some participants responded to questions in a focus group setting while others were in separate interviews is a possible limitation. Had all parties participated in one of the focus groups, their answers may have differed due to peers’ influences. Additionally, answering questions in a group setting allows a singular voice or opinion to monopolize the conversation, potentially differing from the initial thoughts or opinions of the individual participants. The interviewer attempted to mitigate this by allowing time for all participants to answer and even by making eye contact with other individuals. Additionally, asking, “Would anyone else like to contribute to this discussion?” was used to encourage others to participate.

Another possible limitation could include the principal investigator leading the participants. Though the principal investigator was cautious in running the focus groups and interviews, there is always the possibility that participants’ answers could have been led somehow. Acknowledging the fact that the interviewer was a White male with a biracial daughter could have also lent itself to inadvertently leading the participants. Even further, the research team consisted of two Black females and one White male, and this is the lens from which the results were analyzed.

A third limitation was the facilitator’s fear of triggering participants by asking questions about their sexual, smoking, and alcohol behaviors. Because of this, researchers did not collect in-depth information about their health-related behaviors via focus groups or interviews. However, they did use questionnaires to collect this type of data. That being said, another possible limitation is the closed-ended nature of those questions. There was no room available for the respondents to explain why they behaved the way they did, and having that information could have strengthened the results of this research.

Self-selection could be another limitation in this study. Only participants with some intrinsic motivation (a strong feeling about this subject matter) or extrinsic motivation (the gift card participants were given upon completion) would have been inclined to participate. There was a limited sample size of 15 B/MR undergraduate participants from one small liberal arts institution. The number of participants, plus the lack of diverse institutions, could be a limitation for this study. Including HBCUs, State, and Private Institutions could yield different results or magnify the results discovered through this study. As a result, the results are not generalizable to the greater B/MR undergraduate community. Possible biases must be acknowledged as participants who completed the surveys could have spoken individual truths that may not be accurate or applicable to a larger population. Lastly, though measures were taken to prevent this occurrence, there was the potential to miss a theme during the thematic analysis.

## Data Availability

is an option upon request.
